# Identification of Novel Components Influencing Colonization Factor Antigen I Expression in Enterotoxigenic *Escherichia coli*

**DOI:** 10.1371/journal.pone.0141469

**Published:** 2015-10-30

**Authors:** Sara Haines, Sylviane Gautheron, William Nasser, Geneviève Renauld-Mongénie

**Affiliations:** 1 Research Department, Sanofi Pasteur, Marcy-l'Étoile, France; 2 UMR5240 CNRS/INSA/UCB, Université de Lyon 1, Villeurbanne, France, INSA-Lyon, Villeurbanne, France; Centre National de la Recherche Scientifique, Aix-Marseille Université, FRANCE

## Abstract

Colonization factors (CFs) mediate early adhesion of Enterotoxigenic *Escherichia coli* (ETEC) in the small intestine. Environmental signals including bile, glucose, and contact with epithelial cells have previously been shown to modulate CF expression in a strain dependent manner. To identify novel components modulating CF surface expression, 20 components relevant to the intestinal environment were selected for evaluation. These included mucin, bicarbonate, norepinephrine, lincomycin, carbon sources, and cations. Effects of individual components on surface expression of the archetype CF, CFA/I, were screened using a fractional factorial Hadamard matrix incorporating 24 growth conditions. As most CFs agglutinate erythrocytes, surface expression was evaluated by mannose resistant hemagglutination. Seven components, including porcine gastric mucin, lincomycin, glutamine, and glucose were found to induce CFA/I surface expression *in vitro* in a minimal media while five others were inhibitory, including leucine and 1,10-phenanthroline. To further explore the effect of components positively influencing CFA/I surface expression, a response surface methodology (RSM) was designed incorporating 36 growth conditions. The optimum concentration for each component was identified, thereby generating a novel culture media, SP1, for CFA/I expression. CFs closely related to CFA/I, including CS4 and CS14 were similarly induced in SP1 media. Other epidemiologically relevant CFs were also induced when compared to the level obtained in minimal media. These results indicate that although CF surface expression is complex and highly variable among strains, the CF response can be predicted for closely related strains. A novel culture media inducing CFs in the CF5a group was successfully identified. In addition, mucin was found to positively influence CF expression in strains expressing either CFA/I or CS1 and CS3, and may function as a common environmental cue.

## Introduction

Enterotoxigenic *Escherichia coli* (ETEC) are a major cause of diarrhea-associated mortality and morbidity in children under two years of age and travelers to high-risk countries [1,2]. Virulence is principally mediated by the production of colonization factors (CFs) and the heat-labile (LT) and heat-stable (ST) enterotoxins. CFs are a diverse group of proteinaceous surface structures involved in bacterial adhesion in the small intestine, with over 25 described to date, including colonization factor antigen I (CFA/I) and CS1-CS6. They may be present either alone or in combination, with the presence of CS3 commonly associated with either CS1 or CS2 and CS6 associated with CS4 or CS5 [3,4]. The most well-studied CF is the CFA/I fimbriae, which is phylogenetically related to seven other CFs, grouped in the CF5 class [5]. CFA/I, CS4 and CS14 are further grouped in the CF5a subclass, with similar structure and antigenicity [5]. As most CFs are able to bind both intestinal epithelial cells and agglutinate erythrocytes through the same mechanism [6], CF surface expression can be associated with their hemagglutination pattern. Surface expression of many CFs is favored *in vitro* by cultivation in the CFA reference media, originally described for CFA/I [7,8]. However, the CFA media is strikingly different from the intestinal environment to which ETEC is exposed during pathogenesis, making it difficult to determine its relevance to *in vivo* infection.

To date, several intestinal components have been shown to influence CF expression in *in vitro* culture, including bile, glucose, and contact with epithelial cells, though their effects may be CF and/or strain dependent. Bile, for example, is secreted in the proximal small intestine where it facilitates fatty acid digestion and functions as an antimicrobial agent [9]. The sodium glycocholate hydrate and sodium deoxycholate bile salts specifically upregulate CS5 gene expression in a concentration dependent manner [10]. While low concentrations of crude bile salts (0.15%) are also required for surface expression of at least CS8, CS12, CS14, CS17, and CS19, specific bile components have not yet been identified [11,12]. In contrast, expression of CFA/I, CS1-CS4, and CS6 is bile-independent. High glucose concentration inhibits CFA/I gene expression, most likely through a mechanism of catabolite repression [13]. Surprisingly, while glucose has no effect on CS1 or CS3 gene expression in strain E24377A [14], it has been shown to inhibit surface expression of both CS1 and CS3 in strain PB-176 [15]. In contrast, contact with epithelial cells represses gene expression of both CFA/I and CS1 in strains H10407 and E24377A, respectively [16].

Several additional components have also been implicated in CF or toxin expression *in vitro* including temperature, osmolarity, pH, amino acids, iron, zinc, and the lincomycin antibiotic [17–23]. With the exception of temperature, these studies remain isolated, examining the effects of individual components on a limited number of ETEC strains.

Bicarbonate, mucin, ammonium, and signaling molecules such as norepinephrine and cyclic AMP (cAMP) are additional intestinal components that have been shown to modulate virulence in enteropathogens. Bicarbonate is secreted in the proximal small intestine to neutralize gastric acid, and functions as an environmental cue for both *Vibrio cholerae* and *Citrobacter rodentium*, though it has no effect on either CS5 or CS6 in ETEC [10,24,25]. Both gastric and intestinal epithelia are protected from stress by a mucin layer, which undergoes constant renewal and may also serve as a key carbon source for enteric bacteria. Mucin influences both virulence and motility in *Campylobacter jejuni* and enterohemorrhagic *E*. *coli* (EHEC) [26,27]. Furthermore, two proteins have recently been described for their role in mucin degradation by ETEC, indicating that these strains may sense and respond to the presence of mucus [28,29]. Ammonium (NH_4_) inhibits attachment of enteropathogenic *E*. *coli* (EPEC) but favors fimbrial expression in porcine ETEC [30,31]. The neuroendocrine hormone norepinephrine is released under stress conditions, and induces growth and virulence in both porcine ETEC and EHEC [32,33]. Finally, extracellular cAMP has been suggested to function as a signal promoting ETEC adherence to epithelial cells, and acts as an intracellular cofactor for the catabolite repressor protein (CRP) inducing CFA/I expression [34].

Given the diversity of ETEC strains and the lack of identification of components that may modulate global CF expression, we performed a large scale study to evaluate the influence of a variety of components both independently of one another and in combination, with the goal of reproducing a complex culture media reminiscent of the small intestine. Components were selected based on their relevance to the intestinal environment and their known role in modulating enteropathogen virulence, preferably in ETEC. As the majority of studies to date have been performed on CFA/I, and it is one of the most prevalent CFs [3], it was chosen here as an initial model for the response.

Following an initial screening of individual components using a fractional factorial design, those positively influencing CFA/I surface expression were incorporated into a response surface methodology (RSM) to study their combined effect on CFA/I and to evaluate component interactions. Experimental designs are frequently used to optimize culture media with a minimum number of conditions [35,36]. Here, the designs enabled us to determine the relative impact of each component on CFA/I surface expression, identify an optimum condition according to desirability, and appreciate media complexity. To evaluate the diversity of the CF response, surface expression of a variety of CFs was determined in this condition. A novel optimum culture media incorporating six components with relevance to the intestinal environment was successfully identified, inducing CFs within the CF5a subclass.

## Materials and Methods

### Strains and culture conditions

Strains included in this study are described in Table 1. The ETEC strain 258909–3, provided by the Culture Collection at the University of Göteborg, was the principal strain used in the designs and expresses CFA/I. Bacteria were grown in Luria-Bertani broth for routine culture or in M9 minimal salts media (42 mM Na_2_HPO_4_, 22 mM KH_2_PO_4_, 8 mM NaCl, 19 mM NH_4_Cl, 2 mM MgSO_4_, 0.1 mM CaCl_2_, 0.2% (w/v) glucose, pH 7.4) for designs and evaluation of CF surface expression. M9 media was supplemented with the following: Type II porcine gastric mucin, lincomycin hydrochloride, norepinephrine bitartrate salt, 1,10- phenanthroline monohydrate, EGTA, L-glutamine, L-methionine, L-leucine, L-aspartic acid, L-lysine, D-glucose, cAMP, CaCl_2_, ZnCl_2_ (all from Sigma), FeSO_4_, MnCl_2_, bicarbonate (NaHCO_3_), ammonium chloride (NH_4_Cl), and glycerol (all from BDH Prolabo). For comparison, bacteria were cultured in CFA broth, previously described in [7]. 0.15% bile salts (No. 3, Becton Dickenson) were added when necessary for CF expression. All cultures were grown overnight at 37°C with agitation. Culture media and associated components were filter sterilized with the exception of mucin, whose viscosity prevents filtration. Mucin was partially dissolved in deionized water heated to 56°C and autoclaved. The pH of the mucin suspension was adjusted with NaOH as needed and stored for up to four days at 4°C. Media osmolarity was measured before culture with a 210 Micro-osmometer (Fiske).

**Table 1:** Strains used in this study.

Strain	Characteristics	Reference/Origin
258909–3	CFA/I, STh, LT	[37]
H10407	CFA/I, STh, STp, LT	[38]
E24377A	CS1, CS3, STh, LT	[39], NMRC
WS3294A	CS14, ST	NMRC
WS2589A	CS4, CS6, LT, STp	NMRC
E9034A	CS3, CS21, LT, STh	NMRC
LSN03-016011/A	CS17, LT	NMRC
C91f	CS2, CS21, LT, STh	NMRC

### Experimental design matrices

A two-level fractional factorial Hadamard matrix was designed to evaluate the effects of 20 components of interest on ETEC virulence expression. This matrix is used to screen a large number of components and to identify their main effects using the fewest number of experiments, and excludes potential interactions between components. 30 cultures were incorporated, including 24 experimental conditions and six duplicates to evaluate experimental variability (S1 Table). Conditions were evaluated following growth in M9 media diluted two-fold (M9-low) to permit bacterial growth in presence of a variety of components. Each component was tested at two levels: high and low concentration, where the low concentration may be 0. In the design, the high and low levels were coded as 1 and -1, respectively. The design used the following model: Y = b0 + b1 × X1 + b2 × X2 + b3 × X3 + b4 × X4 + b5 × X5 + b6 × X6 + b7 × X7 + b8 × X8 + b9 × X9 + b10 × X10 + b11 × X11 + b12 × X12 + b13 × X13 + b14 × X14 + b15 × X15 + b16 × X16 + b17 × X17 + b18 × X18 + b19 × X19 + b20 × X20 where Y is the endpoint readout (CFA/I surface expression, LT secretion, or bacterial density), b_0_ is the constant term, b_i_ is the linear coefficient and X_i_ is the coded level of the component studied. The model was validated by an analysis of variance (ANOVA), comparing residual error to experimental variability based on the six duplicates (for additional information, see S1 Supplementary Methods). Component effects were tested against the experimental variability and were considered significant when p-values were <5% (<0.05) or at the limit of significance when p-values ranged from 5–10%.

To identify optimum concentrations of components selected from the Hadamard matrix for integration into a single culture media, a customized RSM was generated using a 2-degree polynomial model. The model accounted for the effects of seven components, their square terms, and interactions. 47 cultures were incorporated in the matrix, including 36 experimental conditions, eight test points to validate model quality, and three replicates of the center point to determine experimental variability (S2 Table). Each component was tested at 5 or more levels (S2 Table, S1 Supplementary Methods). The design used the following model: Y = b0 + b1 × X1 + b2 × X2 + b3 × X3 + b4 × X4 + b5 × X5 + b6 × X6 + b7 × X7 + b1-1 × (X1×X1) + b2-2 × (X2×X2) + b3-3 × (X3×X3) + b4-4 × (X4×X4) + b5-5 × (X5×X5) + b6-6 × (X6×X6) + b7-7 × (X7×X7) + b1-2 × (X1×X2) + b1-3 × (X1×X3) + b2-3 × (X2×X3) + b1-4 × (X1×X4) + b2-4 × (X2×X4) + b3-4 × (X3×X4) + b1-5 × (X1×X5) + b2-5 × (X2×X5) + b3-5 × (X3×X5) + b4-5 × (X4×X5) + b1-6 × (X1×X6) + b2-6 × (X2×X6) + b3-6 × (X3×X6) + b4-6 × (X4×X6) + b5-6 × (X5×X6) + b1-7 × (X1×X7) + b2-7 × (X2×X7) + b3-7 × (X3×X7) + b4-7 × (X4×X7) + b5-7 × (X5×X7) + b6-7 × (X6×X7) where Y is the endpoint readout (CFA/I surface expression or bacterial density), b_0_ is the constant term, b_i_ is the regression coefficient and X_i_ is the coded level of the component studied. To check model validity, coefficients were calculated from all experimental conditions, excluding the eight test points. The estimated results determined from the mathematical model in absence of the eight test points were compared to experimental results. The model was validated when test point results corresponded to predicted values, as well as by ANOVA, appreciation of the R^2^, and the distribution of residuals (S1 Supplementary Methods). The effect of each component was tested on the combined variability of both the experimental variability determined from the three center points and the model variability determined from the eight test point conditions for all endpoint responses.

Matrices were generated and analyzed using the NemrodW software v2007. For both matrices, endpoints measured were mannose resistant hemagglutination (MRHA) and bacterial growth based on final culture density at OD_600._ In addition, component effect on LT toxin secretion was evaluated by GM1-ELISA during Hadamard screening. Cultures were performed in the basal M9 media and the CFA reference media in parallel for comparison with experimental conditions.

### Mannose Resistant Hemagglutination (MRHA)

CFs mediate mannose-resistant hemagglutination of erythrocytes. The level of surface expressed CF can therefore be quantified using this functional assay [7]. MRHA was adapted from [40] using human (CFA/I, CS4) or bovine erythrocytes (other CFs). Briefly, bacteria and erythrocytes were washed twice in Dulbecco’s phosphate buffered saline (DPBS) at 4°C and resuspended in cold DPBS containing 0.5% D-mannose (DPBS-M) to a final concentration of 4x10^10^ cfu mL^-1^ for bacteria and 10% for erythrocytes. Erythrocytes were stored for up to 2 weeks at 4°C. Bacteria were serially diluted in 25 μL DPBS-M in 96-well round bottom plates. Erythrocytes were freshly diluted to 0.75% with DPBS-M and 50 μL added to bacterial suspensions. Plates were agitated at 4°C for at least 3 hours before reading the minimal hemagglutination titers (MHT), which remained stable for up to 2 days. Titers were determined according to the number of wells with visible agglutination, and expressed as the log_2_ of the serial dilution for normal distribution.

### GM1 ELISA

LT toxin present in culture supernatant was detected by GM1 ELISA as previously described [41], with quantification against a gradient of purified recombinant LTB (Sigma) at known concentration. Culture supernatant was treated with EDTA-free anti-protease tablets (cOmplete, Roche), filtered at 0.22 μm, and stored at -70°C until analysis by ELISA. Plates were coated with 100 μL GM1 at 0.5 μg mL^-1^ in phosphate buffered saline (PBS) overnight at room temperature, and blocked with 1% bovine serum albumin (BSA) in PBS for 1 hour at 37°C. Culture supernatant was incubated in GM1 coated plates for 1 hour at room temperature for LT binding. Between each step, wells were washed with PBS. LT toxin was then labeled with a rabbit anti-CTB monoclonal antibody (1:5000, Sigma) in Ab buffer (PBS-0.05% Tween (PBS-T) with 0.5% BSA), prior to addition of a goat anti-rabbit IgG secondary antibody coupled to horseradish peroxidase (1:1000, Sigma) in Ab buffer for detection. Incubations were performed at room temperature for 90 min unless otherwise noted and wells washed between each step in PBS-T. Colorimetric detection at 450 nm was performed using 3,3’,5,5’-tetramethylbenzidine and the reaction stopped after 15 min with 1M HCl. In parallel, a gradient of recombinant LTB was labeled, generating a standard curve for titration of secreted LT. Samples were analyzed in duplicate with at least two dilutions. LT toxin levels were normalized against bacterial density at OD_600_.

### Statistical analyses

Spearman’s correlates (ρho) were used to determine statistical dependence between two variables, with ρ > ± 0.7 and a p-value <0.05 indicating a strong correlation (JMP software v7.1). Pairwise comparisons of CF surface expression were performed using the student t-test for parametric data or the Mann-Whitney Rank Sum test for non-parametric data (SigmaPlot 11.0; Systat Software Inc.), with p-values < 0.05 considered significant.

## Results and Discussion

### Development of an experimental design to identify components influencing CFA/I surface expression

For successful colonization of the small intestine, ETEC must be able to sense and respond to changing, and often stressful, environmental conditions. Classically, *in vitro* culture of ETEC is performed in rich, complex media with little-to-no understanding of the specific culture media components mediating virulence factor expression. Although several components are known to play a role in ETEC virulence *in vitro*, including bile salts and glucose, effects are often strain or CF dependent [13–15]. Here, we evaluated the impact of 20 commercially available components to identify those involved in modulating surface expression of the archetype fimbriae CFA/I and to generate a novel culture media with greater relevance to the host intestinal environment. Components were selected based on published data implicating them in virulence factor expression in pathogenic enterobacteria, including ETEC, or their presence in the intestinal environment.

To determine the influence of each component on virulence factor expression and bacterial growth, a Hadamard matrix was designed and 30 experimental conditions tested (S1 Table). The effect of individual components on CFA/I surface expression was determined by MRHA, while effects on the LT toxin were determined by quantification of secreted LT present in the culture supernatant by GM1 ELISA. Component effects on bacterial growth were also determined by measurement of the bacterial density at the end of culture.

The chemically defined M9 minimal salts media containing 0.2% glucose was selected as the basal media for supplementation, as it induced low but detectable levels of CFA/I surface expression and LT secretion in the supernatant (Table 2). Bacterial density in M9 media was also reduced in comparison to that obtained in the CFA reference media generally used to induce CF expression (Table 2). As MRHA and GM1 ELISA analyses require normalization against bacterial quantities, the cfu/OD equivalence was determined at three time points, corresponding to exponential, early stationary, and late stationary growth phases in both CFA and M9 media. No difference could be seen between the two growth conditions (data not shown).

**Table 2:** ETEC virulence response in M9 media as compared to the CFA reference media.

Read-out	CFA	M9	p-val
Surface expressed CFA/I (MHT (log_2_))	7.53 ± 0.30	3.21 ± 0.27	p < 0.001
Secreted LT (ng/OD)	20.8 ± 12.11	2.62 ± 2.29	ns
Bacterial density (OD/mL)	5.27 ± 0.27	1.57 ± 0.20	p < 0.001

CFA and M9 were compared using the Student t-test, p-val is p-value, ns is not significant. Values are ± SEM. MHT: minimal hemagglutination titer

Surprisingly, when the M9 minimal media was supplemented with multiple components, ETEC growth was inhibited in several experimental conditions, possibly due to an increase in culture osmolarity. Indeed culture osmolarity in M9 media was ~220 mOsm/kg but increased to up to 523 mOsm/kg in supplemented cultures. Experiments were therefore performed in M9 media diluted two-fold (M9-low) to permit growth with component supplementation. This allowed us to easily identify components with a positive influence on CFA/I expression, LT secretion, and/or growth.

To evaluate the effect of each component, two inputs were used: exclusion from the culture, or inclusion at a single concentration (S1 Table). As calcium, ammonium, and glucose were present in the basal M9 media, inputs were evaluated with inclusion at two relative concentrations, low and high. End-point analyses were performed to determine the relative impact of each component on CFA/I surface expression, LT secretion and growth. Measurements were performed following overnight culture, as it is optimum for CFA/I surface expression. Despite lower levels of LT toxin production and secretion in stationary phase as compared to exponential phase, trends are similar during both growth phases [19]. Thus, we took advantage of these cultures to evaluate LT secretion in parallel. In addition, initial and final pH were measured, with initial pH set here at 7.4, as described for the CFA reference media [7].

### Identification of environmental components influencing CFA/I surface expression

**Fig 1:**
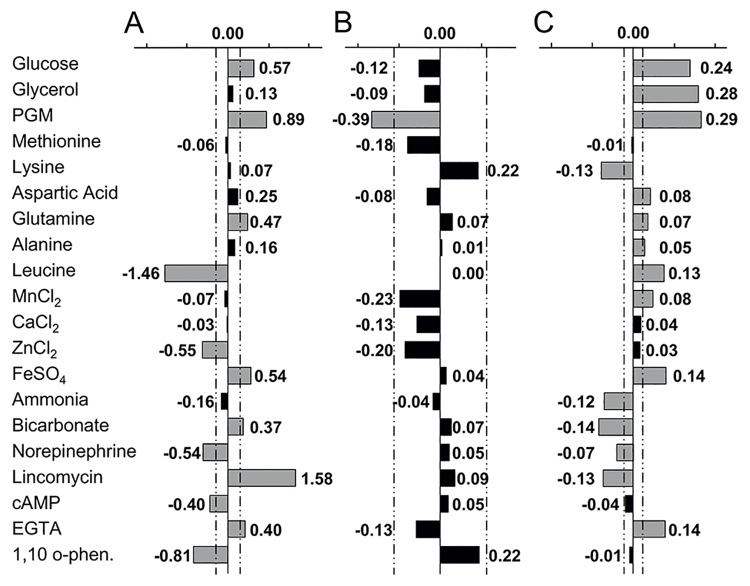
Effect of culture components on CFA/I surface expression, LT secretion, and growth. The effect of individual components on CFA/I surface expression (A), LT secretion (B) and bacterial growth based on final density after overnight culture (C) was determined. The CFA/I expressing strain 258909–3 was cultured overnight in M9-low media supplemented with component combinations defined in the Hadamard matrix. CFA/I surface expression was evaluated by MRHA, LT toxin secretion by ELISA-GM1 of culture supernatant, and growth by OD_600_. Components with a significant effect on a given response are shown in grey and those with no effect in black, with dashed lines representing the threshold of significance for each endpoint (p<5%). Coefficients are indicated for each component and indicate the relative strength of the response. PGM: porcine gastric mucin; 1,10 o-phen: 1,10-phenanthroline.

Of the 20 components evaluated here, seven had a positive influence on CFA/I surface expression while five others had a negative influence (Fig 1A and S3 Table). CFA/I was strongly induced by porcine gastric mucin (PGM) and lincomycin, but strongly inhibited by leucine and the zinc chelator 1,10-phenanthroline. Taken together, these four components account for approximately 75% of the variability of the CFA/I response (S1 Fig).

The most striking result found here was the strong positive effect of PGM. While other fimbriae, including F5 expressed by porcine ETEC, may adhere to decoy ligands present in mucin [42], PGM did not inhibit CFA/I mediated MRHA. Of note, the positive effect of PGM was observed despite previous sterilization by autoclaving, indicating that the original structures of the large, complex glycoproteins found in mucin were not critical to the induction of CFA/I surface expression.

Although MUC2 is the major mucin in the small intestine, PGM is composed mainly of the MUC5AC mucin. While purified mucins specific to the small intestine are not commercially available, PGM may provide a physiologically relevant substitute, inducing a similar response, as both MUC5AC and MUC2 possess similar glycan patterns in the murine model [43]. Furthermore, as the mucus layer undergoes constant renewal, it is likely that neutralized gastric mucins are almost constantly present in the small intestine. Indeed, we found that mucin type influenced the level of CFA/I surface expression. Among the commercially available mucins, type II porcine gastric mucin (PGM) generated a higher level of CFA/I surface expression than bovine submaxillary mucin (BSM) (data not shown) and was therefore selected for the Hadamard matrix. Although the specific sub-component of PGM inducing CFA/I expression has not yet been identified, it does not appear to be sialic acid, as BSM contains 9–17% bound sialic acid, whereas PGM contains only ~1% bound sialic acid (communicated by Sigma). Furthermore, although sialic acid was initially selected for evaluation in this study, it had to be removed from the Hadamard matrix as low levels of sialic acid completely inhibited ETEC growth in minimal media. We postulate that this may be one mechanism by which ETEC senses its location within the gastrointestinal tract, as sialyation is lowest in the distal small intestine which corresponds to the major site of ETEC infection [44], with an increasing concentration gradient throughout the large intestine [45].

Although absent in the small intestine, the antibiotic lincomycin was included in this study as it has been previously described to induce LT toxin synthesis in ETEC [46], and was initially chosen here as a positive control for LT secretion. The positive effect of lincomycin on CFA/I surface expression was unexpected, as subinhibitory concentrations of lincosamides generally inhibit fimbrial production and adhesion [47–49].

In contrast to PGM and lincomycin, leucine had strong inhibitory effect on CFA/I surface expression. Leucine has previously been shown to repress ETEC fimbriae in animal strains, inhibiting expression of the operons encoding CS31A and F5 fimbriae via modulation of the leucine response regulator, Lrp [50,51]. Similarly, the zinc chelator 1,10-phenanthroline inhibited CFA/I surface expression. High zinc concentration inhibits virulence gene expression in EPEC and EHEC, repressing the bundle-forming pilus (*bfp*) and shiga toxin (*stx*) genes, respectively [52,53]. However, at least some zinc may be required for full virulence, as *C*. *jejuni* shows reduced pathogenicity in the chick model in absence of the zinc transporter, ZnuA [54]. Similarly, LT toxin secretion may be increased in the presence of zinc [22]. Although CFA/I was inhibited by 1,10-phenanthroline, it was also inhibited in presence of 25 μM excess zinc. This apparent contradiction may be explained by an indirect effect of 1,10-phenanthroline, chelating another metal cation, or the need for an intermediate zinc concentration for optimum CFA/I expression.

While bicarbonate has no effect on the expression of either CS5 or CS6 [10], it has been identified as a signal for virulence in several enteropathogens [24,25]. Bicarbonate had a positive effect on CFA/I surface expression here, although the response was weak (Fig 1A). However, as bicarbonate is interchangeable with CO_2_, this may be due to the use of oxygenated media which can mimic the bicarbonate effect by generating higher basal CO_2_ levels. Bicarbonate may therefore induce a greater effect on CFA/I surface expression when tested in low-oxygen conditions.

Despite earlier studies showing that high glucose and iron concentration inhibit CFA/I expression [13,21], both components were found to induce CFA/I surface expression in the conditions tested here. Of note, glucose inhibited *cfaA* promoter activity in the SP1 media (data not shown), confirming that this operon is subject to catabolite repression. However, this inhibition had no subsequent effect on CFA/I as determined by MRHA, suggesting that ETEC incorporates additional signals at the post-transcriptional level for fimbrial regulation and surface expression.

Glutamine and aspartic acid also induced surface expression of the CFA/I fimbriae, as did the presence of EGTA (Fig 1A). The positive effect of the EGTA calcium chelator suggests that CaCl_2_ may negatively influence CFA/I surface expression, as it was present in the basal media. However, supplementation with even higher CaCl_2_ concentrations (900 μM) had no significant negative effect.

In contrast to CFA/I, no components were identified with a significant positive influence on LT toxin secretion and only a single component, PGM, had a negative effect (Fig 1B and S3 Table). We initially postulated that PGM might function as an environmental cue inhibiting LT toxin expression or synthesis. However, further studies showed that incubation of recombinant LT toxin (rLTB) in culture media containing PGM prevented its detection by GM1 ELISA in a concentration dependent manner, whereas it was detected in absence of PGM with no degradation (S2 Fig). A similar effect of PGM has been previously reported for the cholera toxin [55]. As PGM was autoclaved before use, it does not retain enzymatic capacity associated with protein degradation. Thus PGM most likely masked the LT toxin epitope mediating binding to the GM1 receptor.

Of note, despite a strong inhibition of secreted LT by PGM, secreted LT levels were also low (<1 ng/OD) in the absence of PGM. Low toxin secretion is a characteristic of the CFA/I expressing strain used in this study, as seen in the M9 media, with further inhibition of detection due to the presence of PGM in many experimental conditions (S1 Table).

Although lincomycin has been reported to induce LT secretion in the CFA/I expressing strain H10407 [46], it had no effect on LT secretion in the experimental conditions tested here. While the effect of lincomycin may be strain specific, leading to increased production of other virulence factors including CFA/I, as shown here for strain 258909–3, lincomycin has also been reported to induce LT production with no associated increase in secretion [56].

As expected, bacterial growth was strongly induced in the presence of glucose and glycerol, which are known carbon sources for *E*. *coli*, as well as in the presence of PGM (Fig 1C and S3 Table). The addition of individual amino acids had a variable effect on growth. Aspartic acid and glutamine enhanced growth, as did leucine and alanine. In contrast, lysine inhibited growth whereas methionine had no effect. FeSO_4_ and ETGA induced growth in addition to CFA/I surface expression, while MnCl_2_ positively influenced growth alone. Of note, MnCl_2_ also enhances bacterial growth in the CFA reference media though it has no effect on CFA/I surface expression. In contrast to other enteropathogens, norepinephrine inhibited bacterial growth in ETEC [33,57], as did bicarbonate, lincomycin and a high ammonium concentration (50 mM).

As pH was not buffered in these media, the final pH varied from 4.9 to 8.9. The final pH was not correlated with CFA/I surface expression or LT toxin secretion, but was negatively correlated with bacterial density (ρho = -0.37, p = 4.6%) (S4 Table). However, based on the weak correlate (ρho<0.7), this effect was not considered to be significant.

### Effect of porcine gastric mucin on CF expression in ETEC strains H10407 and E24377A

PGM strongly induced CFA/I surface expression, as determined by the Hadamard matrix. Given the importance of mucin in the small intestine, we hypothesized that this component might represent a global environmental cue for CF production. Previously, CFA/I and CS1/CS3 (expressed by strains H10407 and E24377A, respectively) were shown to respond differentially to both bile and glucose [13–15]. For comparison, we evaluated the effect of PGM on CF surface expression of these two strains, as determined by their ability to agglutinate erythrocytes (MRHA).

**Fig 2:**
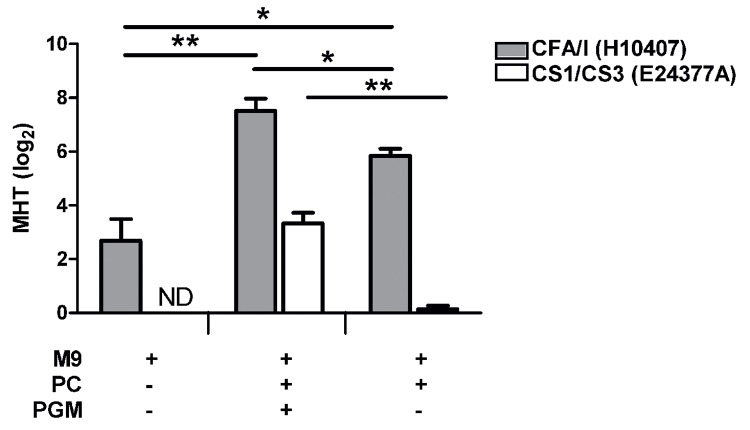
Effect of PGM on surface expressed CFA/I or CS1/CS3. ETEC strains expressing CFA/I (H10407) or CS1/CS3 (E24377A) were cultured in M9 media alone or supplemented with all components found to have a positive effect (p<10%) on CFA/I via the Hadamard matrix including glucose, aspartic acid, glutamine, FeSO_4_, bicarbonate, lincomycin and EGTA (PC) in the presence or absence of porcine gastric mucin (PGM). MRHA was performed following overnight growth, with the minimal hemagglutination titer (MHT) expressed in log_2_. Means of at least two independent replicates are shown with error bars representing the standard error of the mean (SEM). Statistical analyses were performed using the Mann-Whitney test, * p<0.05, ** p<0.01. ND: not detected.

CF expression was increased following bacterial culture in presence of all positive components identified by the Hadamard matrix when compared to M9 media alone for both strains (Fig 2). This indicated that the overall positive effect of components identified in the Hadamard matrix for CFA/I was also maintained for CS1/CS3. When PGM was removed from culture media, the minimal hemagglutination titer (MHT) was inhibited for H10407, confirming its positive effect on CFA/I (p<0.05). PGM also significantly induced CS1/CS3 surface expression of strain E24377A, as removal of PGM from media completely abolished agglutination in this strain (p<0.01). This indicates that the main factor mediating surface expression of CS1/CS3 is PGM in this condition.

### Optimization of a culture media inducing CFA/I surface expression

Next, components positively influencing CFA/I surface expression were downselected for further optimization. In addition to establishing individual optimum concentrations, their concomitant optimization enabled us to identify a novel culture media inducing CFA/I surface expression with relevance to the intestinal environment. Six factors, including PGM, glucose, glutamine, lincomycin, EGTA, and FeSO_4_ were downselected. Aspartic acid was not retained here as its effect was at the limit of significance (p = 6.4%, S3 Table). Similarly, bicarbonate was eliminated due to its strong inhibitory effect on bacterial growth. An RSM design incorporating 47 experimental conditions was used to determine the optimum concentration for each component, and to identify possible interactions between components which could influence the response (S2 Table). The effect of the initial pH was also evaluated at this stage, as it can modulate both fimbrial and toxin expression [19,31] and is a key intestinal factor.

**Fig 3:**
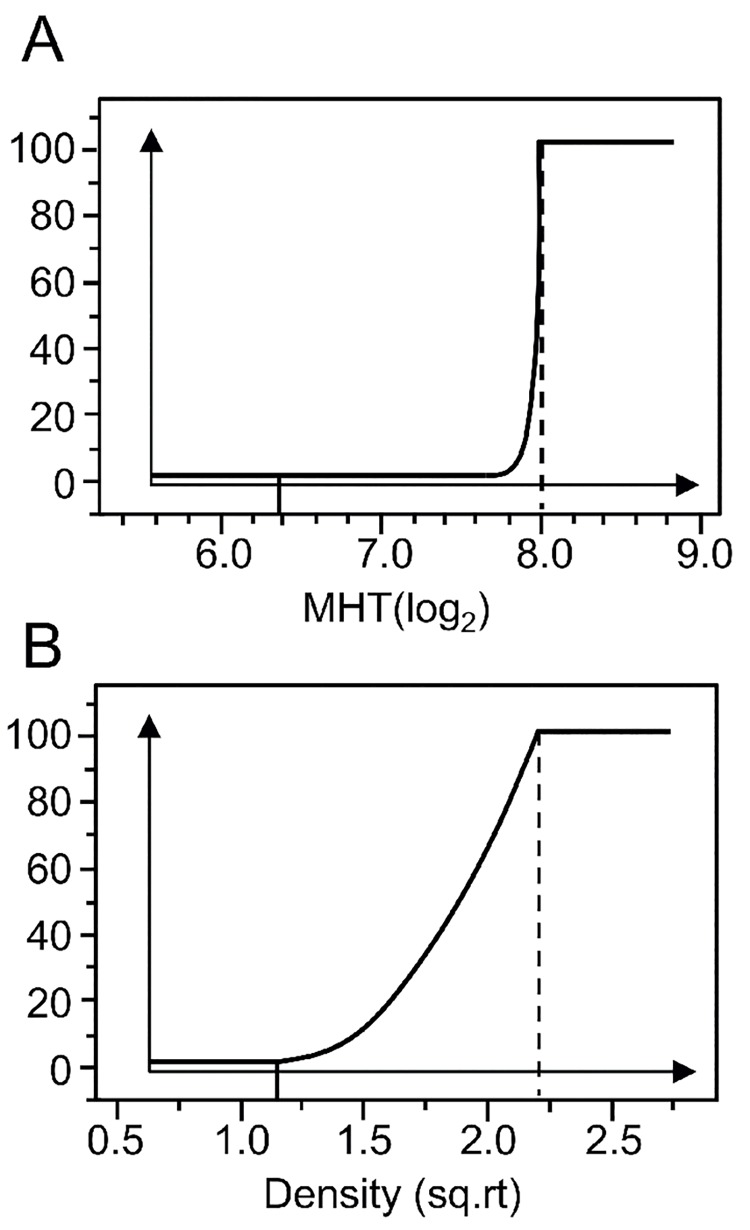
Desirability curves for identification of an optimum condition. Desirability curves were established for (A) CFA/I surface expression (minimal hemagglutination titers (MHT) determined by the MRHA assay) and (B) bacterial density (OD_600_) with desirability ranging from 0–100%. For normal distribution, MHT titers are transformed in log_2_ and growth is transformed as a square root (sq.rt.).

To identify the optimum concentration for each component, desirability curves were defined (Fig 3). The optimum concentration was required to generate a higher response than that obtained in the M9 basal media and an equivalent or higher response when compared to the CFA reference media. As the principal goal in culture media optimization was to favor CFA/I surface expression, desirability was most restricted for the MRHA assay, with 100% desirability above an MHT of 8. In contrast, desirability increased over a gradient for bacterial growth. Given the low levels of secreted LT toxin seen previously, and the incorporation of PGM into all experimental conditions in the RSM, secreted LT toxin was not evaluated here.

An optimum culture media was successfully identified for all endpoints based on the desirability curves and was designated SP1. Variation limits around the optimum were estimated for each component and indicate the range for which a maximum response in CFA/I surface expression should still be attained (Table 3). Overall, variation limits were restrained, with differences between the minimum and maximum values of less than two-fold. Optimum PGM concentration was predicted to be superior to the experimental range, at 2.1g/L, with the model simulating the response for up to 2.6 g/L. Optimum pH was ranged from 6.8–7.7, corresponding closely to intraluminal pH levels encountered within the small intestine (~6.6–7.5) [58]. The CFA reference media has an initial pH of 7.4, similar to the optimum obtained here (pH 7.5).

**Table 3:** Identification of an optimum condition for CFA/I surface expression using response surface methodology.

Factor	Tested Range	Theoretical range of model	Optimum condition	Variation limits	Unit
PGM	0.1–2.0	0–2.6	2.1	1.3–2.2	g/L
Glucose	5.5–55	0–66.7	44.4	22.2–44.4	mM
Glutamine	0.34–13.7	0–16.4	10.3	6.18–11.0	mM
Lincomycin	10–90	0–102	74.7	67.2–74.8	μg/mL
EGTA	0.1–1.0	0–1.2	0.5	0.5–0.6	mM
FeSO_4_	5–200	0–228.5	73.4	70.1–87.4	μM
pH	6.0–8.5	5.2–9.3	7.5	6.8–7.7	-

Based on the RSM, theoretical endpoint values were determined for the optimum condition prior to experimental evaluation (Table 4). Bacterial culture in the SP1 media generated comparable levels of CFA/I surface expression but higher bacterial density and final pH when compared to predicted values. Comparison of the SP1 media with the CFA reference media showed no significant difference in CFA/I surface expression, while bacterial density was significantly higher in SP1 media (p<0.001) (Tables 2 and 4).

**Table 4:** Predicted and experimental read-outs of bacterial culture in SP1 media based on the response surface methodology.

Read-out	Predicted Value	Experimental Value ± SEM
Surface expressed CFA/I (MHT (log_2_))	8.97	7.75 ± 0.89
Bacterial density (OD/mL)	5.76	9.02 ± 0.43
Final pH	5.72	6.93 ± 0.15

Experimental value is an average of at least 6 replicates

Component effects on CFA/I surface expression and growth were evaluated in both linear and square terms, with interactions between individual components determined for both endpoints (S5 Table). Squared functions of both pH and glutamine significantly influenced CFA/I surface expression, indicating that the optimum corresponded to an intermediate point within a bell curve (S5 Table). Similarly, squared functions of mucin, glucose and pH influenced bacterial growth. Despite the incorporation of only seven components, a large number of interactions significantly influenced CFA/I surface expression, providing an indication of the complexity of the virulence response in this culture media. With the exception of glucose, all other components interacted. pH, lincomycin, and glutamine each interacted with three other components, while EGTA interacted only with PGM (S5 Table). Of note, the interaction between mucin and glutamine was associated with CFA/I surface expression, with higher levels of both components inducing MRHA (S3 Fig). This may be relevant *in vivo*, as glutamine is associated with intestinal integrity and itself induces mucin expression by goblet cells [59]. In contrast to CFA/I surface expression, lincomycin and pH were the two main components involved in interactions influencing bacterial growth. Notably, all interactions including lincomycin inhibited growth, as did lincomycin alone, indicative of its bacteriostatic effect.

Given the large number of interactions observed here in the SP1 media, complex media such as CFA are likely subject to many more component interactions. Clearly, these must be taken into consideration when attempting to evaluate the role of specific components on ETEC virulence and growth.

### Effect of optimized culture media on surface expression of other CFs

To determine if other epidemiologically relevant CFs could be induced in the optimized SP1 media [60], additional ETEC strains expressing CFA/I, CS4, CS14 (CF5a family), CS1, CS2, CS3, or CS17 were analyzed by MRHA. Inclusion of a second CFA/I expressing strain (H10407) was performed to verify that the SP1 media was not strain-specific. As certain CFs require the presence of bile salts, they were incorporated into the SP1 media as needed. In parallel, CF surface expression was evaluated in both the basal M9 media and the CFA reference media, supplemented or not with bile salts.

**Fig 4:**
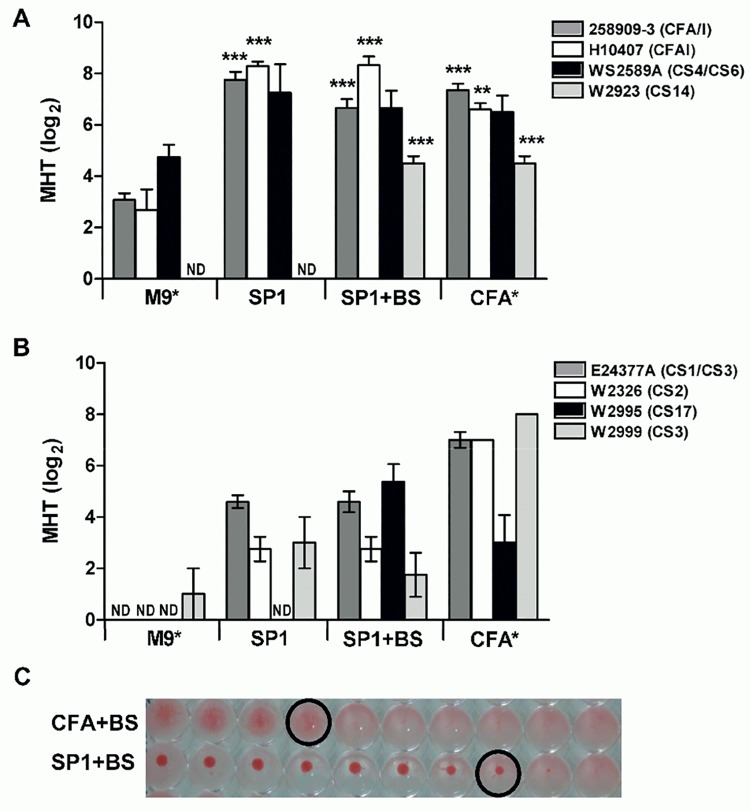
Evaluation of CF surface expression following growth in optimized culture media SP1. CF surface expression was evaluated by MHRA in ETEC strains following overnight growth in M9, SP1, or CFA media in the absence or presence of 0.15% bile salts (BS). CF surface expression was evaluated in strains expressing fimbriae within the CF5a family (A) or other CFs (B). Data are expressed as the minimal hemagglutination titer (MHT) required for agglutination of human or bovine erythrocytes transformed in log_2_ for normal distribution. ND indicates not detected. The hemagglutination pattern of CS17-expressing strain is shown in (C). *Strains expressing CS14 or CS17 were cultivated in M9 or CFA media supplemented with BS, while all other strains were cultivated in M9 or CFA in the absence of BS. Data are shown as average of at least three independent replicates, with bars representing the SEM. Statistical analyses were performed using the Mann Whitney test (A), with surface expression in SP1 and CFA media compared to basal M9 media. *p<0.01, ** p<0.001.

CFs within the CF5a family (CFA/I, CS4, CS14) all showed higher surface expression in the SP1 media than in the M9 basal media and equivalent or higher MHT titers when compared to the CFA reference media (Fig 4A). As expected, CS14 required the presence of bile salts; no agglutination could be seen in SP1 media in the absence of bile despite the presence of a variety of additional components. However, the addition of bile salts alone was not sufficient to induce CS14 in M9 media, suggesting that other components are also required. The MRHA response measured in strain WS2589A (CS4/CS6) was specific to the CS4 fimbriae as CS6 alone does not agglutinate red blood cells [61].

Other CFs, including CS1, CS2, CS3 and CS17 also showed higher surface expression in SP1 media than in the basal M9 media (Fig 4B). Indeed, no hemagglutination could be seen in M9 media for strains expressing CS1, CS2, or CS17. Bile salts had no effect on MRHA mediated by CS1/CS3 or CS2 as expected [11], but were required for expression of CS17. Surprisingly, bile salts inhibited CS3-mediated agglutination in a strain expressing only CS3 by nearly two titers. In contrast to CF5a strains, CF surface expression in the SP1 media did not reach levels comparable to those seen in the CFA reference media, with the exception of CS17. This result was confirmed with two distinct strains expressing CS17 (data not shown). In addition to having stronger titers, CS17-mediated agglutination was complete following growth in SP1 media, whereas it was partial and diffuse following growth in CFA (Fig 4C).

## Conclusion

In summary, the optimized SP1 culture media, incorporating six components with relevance to the small intestine, successfully induced CFA/I surface expression to levels similar to those obtained in CFA. Furthermore, results from these experimental designs indicate that it is possible to predict responses of closely related CFs. While CFs in the CF5a subfamily responded in a similar manner to the archetype fimbriae CFA/I, other subgroups within the CF5 family were not induced to the same extent. These data confirm recent studies [10,14] indicating that different CFs respond to different components, or at the very least to different component concentrations.

Taken globally, these data indicate that virulence cues are complex and interdependent, even in *in vitro* culture. However, the use of an experimental design allowed us to determine the relative impacts of a variety of components on virulence factor expression and to appreciate component interactions. Here, we have shown that ETEC uses a variety of external cues to modulate at least CFA/I surface expression, several of which are present in the distal small intestine, including glutamine, mucin, and a pH near 7.5.

On a larger scale, it seems evident that ETEC must respond to a hierarchy of signals to sense arrival in the appropriate environment and to modulate virulence, with less critical roles for glucose and iron in the presence of other components. Despite the identification of 12 components modulating the CFA/I response, additional variables present in the small intestine, including antimicrobial peptides, gastric hormones, and the gut microbiota, remain to be explored.

## Supporting Information

S1 Fig Component effect on the variability of the CFA/I response. Pareto distribution diagram showing the percent impact of each component on CFA/I surface expression.
(PDF)

S2 Fig Concentration dependent LTB epitope masking by PGM. 20 ng/mL recombinant LTB (rLTB) was incubated in SP1 culture media containing 0 to 2100 mg/L PGM for 1 hour before LTB quantification by GM1 ELISA.
(PDF)

S3 Fig Effect of the interaction between glutamine and PGM on the CFA/I response. 2-dimensional (A) and 3-dimensional (B) representation of the interaction between glutamine (mM) and PGM (g/L) on CFA/I surface expression as determined by MRHA. MHT titers are indicated by blue lines (A) or color code (B), with the optimum corresponding to the highest titer induced by the interaction.
(PDF)

S1 Supplementary Methods (PDF)

S1 Table Hadamard matrix with endpoints. (PDF)

S2 Table Response surface matrix with endpoints. (PDF)

S3 Table Significance and deviation of component effects on endpoints as determined by the Hadamard matrix. (PDF)

S4 Table Endpoint association with final pH using Spearman’s correlates. (PDF)

S5 Table Evaluation of component effects and interactions in SP1 media on CFA/I expression and bacterial density. (PDF)
