# Supplementary material for: Identification of Novel Components Influencing Colonization Factor Antigen I Expression in Enterotoxigenic Escherichia coli
Source: PLoS One. 2015 Oct 30;10(10):e0141469. doi: 10.1371/journal.pone.0141469 (PMC4627747; doi:10.1371/journal.pone.0141469)
Supplement: S1 Fig — Pareto distribution diagram showing the percent impact of each component on CFA/I surface expression. (PDF) [file pone.0141469.s001.pdf]

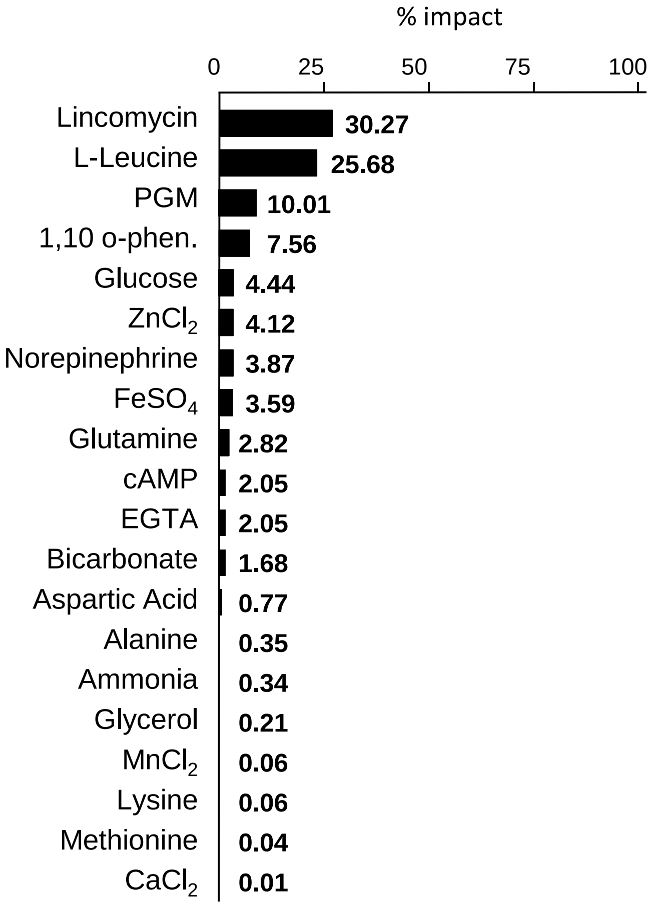

Figure S1. Component effect on the variability of the CFA/I response. Pareto distribution diagram showing the percent impact of each component on CFA/I surface expression.
