# Supplementary material for: Identification of Novel Components Influencing Colonization Factor Antigen I Expression in Enterotoxigenic Escherichia coli
Source: PLoS One. 2015 Oct 30;10(10):e0141469. doi: 10.1371/journal.pone.0141469 (PMC4627747; doi:10.1371/journal.pone.0141469)
Supplement: S2 Fig — 20 ng/mL recombinant LTB (rLTB) was incubated in SP1 culture media containing 0 to 2100 mg/L PGM for 1 hour before LTB quantification by GM1 ELISA. (PDF) [file pone.0141469.s002.pdf]

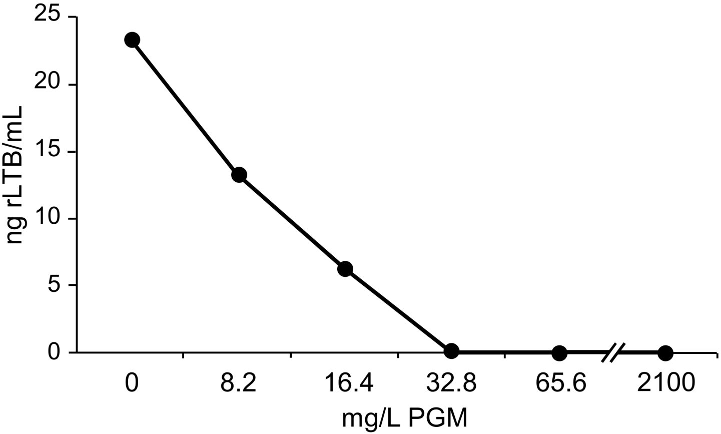

Figure S2. Concentration dependent LTB epitope masking by PGM. 20 ng/mL recombinant LTB (rLTB) was incubated in SP1 culture media containing 0 to 2100 mg/L PGM for 1 hour before LTB quantification by GM1 ELISA.
