# Supplementary material for: Identification of Novel Components Influencing Colonization Factor Antigen I Expression in Enterotoxigenic Escherichia coli
Source: PLoS One. 2015 Oct 30;10(10):e0141469. doi: 10.1371/journal.pone.0141469 (PMC4627747; doi:10.1371/journal.pone.0141469)
Supplement: S3 Fig — 2-dimensional (A) and 3-dimensional (B) representation of the interaction between glutamine (mM) and PGM (g/L) on CFA/I surface expression as determined by MRHA. MHT titers are indicated by blue lines (A) or color code (B), with the optimum corresponding to the highest titer induced by the interaction. (PDF) [file pone.0141469.s003.pdf]

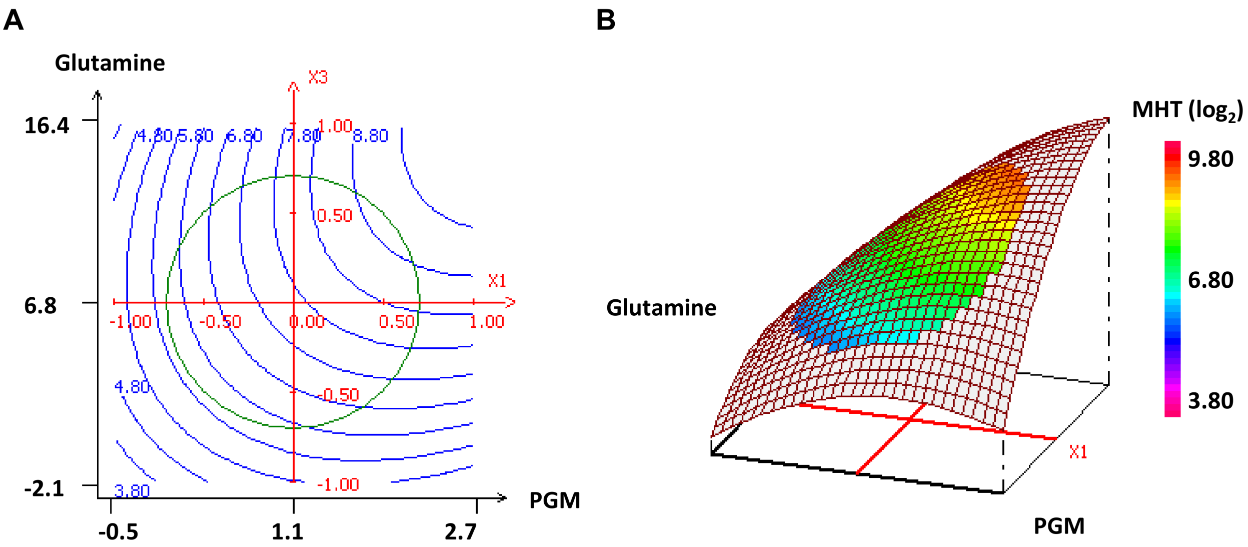


**Figure S3. Effect of the interaction between glutamine and PGM on the CFA/I response.** 2-dimensional (A) and 3-dimensional (B) representation of the interaction between glutamine (mM) and PGM (g/L) on CFA/I surface expression as determined by MRHA. MHT titers are indicated by blue lines (A) or color code (B), with the optimum corresponding to the highest titer induced by the interaction.
