# Supplementary material for: Identification of Novel Components Influencing Colonization Factor Antigen I Expression in Enterotoxigenic Escherichia coli
Source: PLoS One. 2015 Oct 30;10(10):e0141469. doi: 10.1371/journal.pone.0141469 (PMC4627747; doi:10.1371/journal.pone.0141469)
Supplement: S1 Supplementary Methods — (PDF) [file pone.0141469.s004.pdf]

Supplementary methods S1

Hadamard experimental design matrix

The Hadamard matrix model was validated by comparing test point results to predicted values using a student t-test. This test should yield a non-significant response, as actual test points should be similar to predicted points in a robust model (p=8.4). Model quality was also determined by ANOVA, which provided the R^2 coefficient. This coefficient ranges from 0 to 1, with higher R^2 values associated with stronger models. An acceptable cutoff for the R^2 is 0.75. For the MHT endpoint, the R^2 coefficient was 0.990 indicating that the model could explain 99% of the variation in the response. Finally, the model was validated based on the distribution of residuals, which should have a random distribution and be located near the 0 point of the y-axis, though acceptable ranges are generally from -2 to 2.

Analysis of variance (ANOVA) of the MHT endpoint

Source of variation	Sum of squares	Degrees of freedom	Mean squares	Ratio	p-val
Regression	227.51	20	11.38	39.86	< 0.01
Residual	2.28	8	0.29		
Validity	1.28	2	0.64	3.85	8.4
Error	1.00	6	0.17		
Total	229.79	28			

R^2: 0.990; Mean squares = Sum of squares/Degrees of freedom; p-val <0.1 are considered significant

Residual distribution of the MHT endpoint

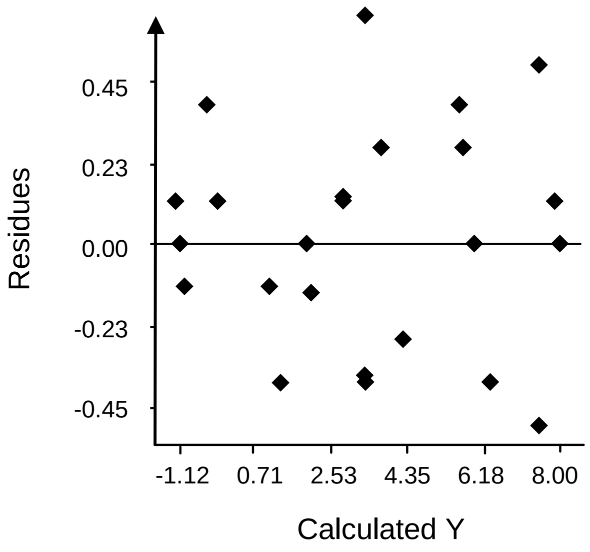

Prior to analysis, endpoints were transformed to yield normal distribution and null values replaced with a minimum value for inclusion. MHT titers were previously transformed in $\log_2$, and were not subject to further modification. In absence of agglutination, null values were replaced by half of the first dilution, or 0.5. Secreted LT (pg/OD) was transformed in $\log_{10}$. When toxin levels were undetected, the null value was replaced by half of the first standard dilution, or 7.8 pg/mL, prior to normalization against bacterial density. Bacterial density (OD/mL) was square root transformed. In absence of bacterial growth, a threshold of 0.01 was applied, corresponding to the density of the inoculum.

Response Surface Methodology (RSM)

As described for the Hadamard matrix, the RSM model was validated by comparing test point results to predicted values using a student t-test, which yielded non-significant results. ANOVA, R^2, and the distribution of residuals were also used to validate the model as described above. Following validation, the mathematical model was then used to predict which variable levels generated the maximum Y endpoint response. The tested and coded levels used for each variable evaluated in the RSM are also included below.

Significance of the eight test points when compared to the model of the MHT endpoint

Experiment	Experimental Y	Calculated Y	Difference	t.exp.	dU	Degrees of Freedom	Standard Deviation	p-val (%)
1	7.000	6.662	0.338	0.974	0.220	3	0.347	40.2
2	7.750	7.270	0.480	1.381	0.220	3	0.347	26.1
8	6.750	6.988	-0.238	-0.685	0.219	3	0.347	54.2
19	7.500	6.782	0.718	2.067	0.219	3	0.347	13.1
22	6.750	6.905	-0.155	-0.445	0.220	3	0.347	68.6
28	8.000	7.718	0.282	0.811	0.221	3	0.348	47.7
33	6.000	7.068	-1.068	-3.072	0.221	3	0.348	5.4
46	7.500	7.056	0.444	1.271	0.232	3	0.349	29.3

Analysis of variance (ANOVA) of the MHT endpoint, including test points

Source of variation	Sum of squares	Degrees of freedom	Mean squares	Ratio	p-val
Regression	46.8218	35	1.3378	6.0668	0.146 **
Residual	2.4256	11	0.2205		
Validity	2.2589	9	0.2510	3.0119	27.4
Error	0.1667	2	0.0833		
Total	49.2473	46			

R^2: 0.951; Mean squares = Sum of squares/Degrees of freedom; p-val <0.1 are considered significant

Residual distribution of the MHT endpoint

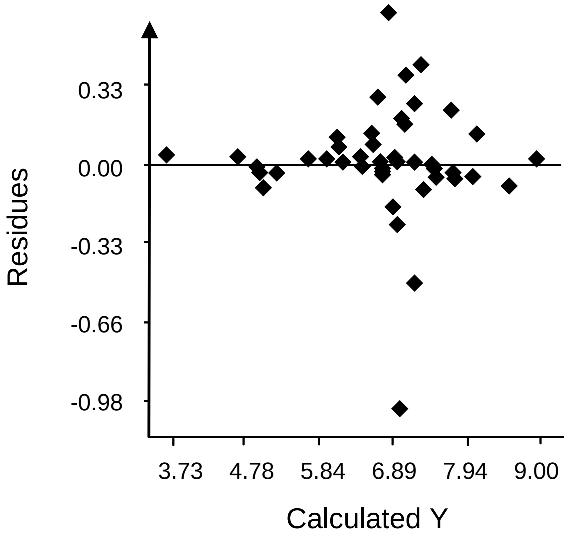

Components evaluated in the RSM with tested and coded levels

PGM		Glucose		Glutamine		FeSO4		Lincomycin		EGTA		pH	
**T (g/L)**	**X**	**T (%)**	**X**	**T (mM)**	**X**	**T (μM)**	**X**	**T (μg/mL)**	**X**	**T (mM)**	**X**	**T**	**X**
0.1	-0.61	0.1	-0.71	0.5	-0.75	5.0	-0.77	10.0	-0.79	0.1	-0.80	6.0	-0.60
0.4	-0.38	0.3	-0.34	4.0	-0.48	55.4	-0.37	34.8	-0.31	0.4	-0.26	7.1	-0.07
1.1	0	0.4	-0.22	7.2	-0.24	78.9	-0.19	42.7	-0.15	0.6	0	7.3	0
1.7	0.38	0.6	0	8.4	-0.15	87.4	-0.12	45.5	-0.10	0.7	0.13	8.3	0.50
2.0	0.61	0.8	0.35	10.4	0	102.5	0	50.5	0	0.9	0.50	8.5	0.60
		1.0	0.68	13.7	0.25	126.9	0.19	58.6	0.16	1.0	0.65		
				16.5	0.46	162.7	0.48	75.5	0.49				
				16.8	0.48	173.2	0.56	81.8	0.61				
				20.0	0.72	200.0	0.75	89.7	0.76				

Components evaluated in the RSM, also shown in Table S2, are shown above at tested (T) levels and at the corresponding coded levels (X) used in the mathematical model.
