# Supplementary material for: Identification of Novel Components Influencing Colonization Factor Antigen I Expression in Enterotoxigenic Escherichia coli
Source: PLoS One. 2015 Oct 30;10(10):e0141469. doi: 10.1371/journal.pone.0141469 (PMC4627747; doi:10.1371/journal.pone.0141469)
Supplement: S1 Table — (PDF) [file pone.0141469.s005.pdf]

Table S1: Hadamard matrix with endpoints

	Components tested																				Endpoint results			
Experiment	Glucose %	Glycerol %	PGM g/L	Methionine g/L	Lysine g/L	Aspartic Acid g/L	Glutamine g/L	Alanine g/L	Leucine g/L	$MnCl_2$ µM	$CaCl_2$ µM	$ZnCl_2$ µM	$FeSO_4$ µM	NH_4Cl mM	$NaHCO_3$ µM	Norep. µM	Lincomycin µg/mL	cAMP µM	EGTA mM	1, 10 o-phen. µM	MHT (log_2)*	Secreted LT (pg/OD)	Bacterial density (OD/mL)	Final pH
1	0.5	0	1	1	0	0	1	1	0	40	50	25	0	9.5	0	0	45	50	0.5	5	6	3.49	4.56	7.56
2[a]	0.5	0.2	1	1	1	0	1	0	1	0	50	25	25	9.5	0	25	0	0	0	0	2	46.24	6.70	6.71
3	0.1	0.2	0	1	1	0	0	1	1	0	900	0	25	9.5	0	0	0	50	0.5	5	0	226.19	2.90	6.69
4[b]	0.1	0.2	0	0	0	0	1	1	1	40	50	25	0	50	22.5	0	0	50	0	0	0	45.24	1.26	7.77
5	0.5	0.2	0	0	1	1	0	0	1	40	50	0	0	9.5	22.5	25	45	50	0	5	1	357.89	1.66	6.67
6	0.5	0.2	1	1	0	1	0	1	1	0	900	25	0	9.5	22.5	0	45	0	0	0	6	9.76	6.27	7.08
7[c]	0.1	0	1	1	0	0	1	0	1	0	50	0	25	50	22.5	25	45	50	0	5	3	146.90	0.83	8.34
8	0.5	0	0	1	1	0	0	1	0	0	50	0	0	50	22.5	25	45	0	0.5	0	0	NA	0.00	NA
9	0.1	0.2	1	1	1	1	0	1	0	40	50	0	25	50	0	0	45	50	0	0	8	19.18	3.20	7.75
10	0.5	0.2	0	1	0	1	1	0	0	40	50	0	25	9.5	22.5	0	0	0	0.5	5	6	64.10	5.48	6.50
11	0.5	0.2	0	0	1	0	1	0	0	0	900	25	25	50	22.5	0	45	50	0.5	0	8	42.58	2.60	6.37
12	0.1	0.2	0	1	0	0	0	0	1	40	900	25	0	50	0	25	45	0	0.5	5	0	16.00	2.35	6.19
13	0.1	0	0	0	0	0	0	0	0	0	50	0	0	9.5	0	0	0	0	0	0	2	97.14	0.77	7.12
14[d]	0.1	0.2	1	0	0	1	1	0	0	0	900	0	0	9.5	0	25	45	50	0.5	0	8	29.47	3.40	7.60
15[a]	0.5	0.2	1	1	1	0	1	0	1	0	50	25	25	9.5	0	25	0	0	0	0	2	10.83	6.65	6.75
16	0.1	0.2	1	0	0	1	0	1	0	0	50	25	25	50	22.5	25	0	0	0.5	5	3	12.60	4.05	8.66
17	0.5	0	0	0	0	1	1	1	1	0	900	0	25	50	0	0	45	0	0	5	4	636.60	2.54	6.32
18	0.1	0	0	1	1	1	1	1	0	0	900	25	0	9.5	22.5	25	0	50	0	5	0	503.67	0.28	8.03
19[e]	0.5	0.2	1	0	1	0	1	1	0	40	900	0	0	50	0	25	0	0	0	5	4	218.09	4.59	6.12
20[b]	0.1	0.2	0	0	0	0	1	1	1	40	50	25	0	50	22.5	0	0	50	0	0	0	149.34	1.91	7.22
21	0.1	0	0	0	1	1	1	1	1	40	50	25	25	9.5	0	25	45	0	0.5	0	4	73.42	2.23	8.36
22	0.5	0	1	0	0	0	0	1	1	40	900	0	25	9.5	22.5	25	0	50	0.5	0	3	8.40	6.34	7.93
23	0.1	0	1	1	1	1	1	0	1	40	900	0	0	50	22.5	0	0	0	0.5	0	3	0.00	2.37	8.79
24[f]	0.1	0	1	0	1	0	0	0	0	40	900	25	25	9.5	22.5	0	45	0	0	5	6	119.47	0.84	8.41
25	0.5	0	0	1	0	1	0	0	0	40	900	25	25	50	0	25	0	50	0	0	1	0.00	1.66	4.90
26[f]	0.1	0	1	0	1	0	0	0	0	40	900	25	25	9.5	22.5	0	45	0	0	5	6	84.48	0.76	8.58
27	0.5	0	1	0	1	1	0	0	1	0	50	25	0	50	0	0	0	50	0.5	5	0	54.55	3.40	7.04
28[c]	0.1	0	1	1	0	0	1	0	1	0	50	0	25	50	22.5	25	45	50	0	5	3	205.34	0.82	8.36
29[d]	0.1	0.2	1	0	0	1	1	0	0	0	900	0	0	9.5	0	25	45	50	0.5	0	7	34.31	3.53	7.69
30[e]	0.5	0.2	1	0	1	0	1	1	0	40	900	0	0	50	0	25	0	0	0	5	3	3.93	4.18	6.90

[a-f] indicate duplicates

*MHT indicates minimal hemagglutination titer detected by MRHA
