# Supplementary material for: Identification of Novel Components Influencing Colonization Factor Antigen I Expression in Enterotoxigenic Escherichia coli
Source: PLoS One. 2015 Oct 30;10(10):e0141469. doi: 10.1371/journal.pone.0141469 (PMC4627747; doi:10.1371/journal.pone.0141469)
Supplement: S2 Table — (PDF) [file pone.0141469.s006.pdf]

Table S2. Response surface matrix with endpoints

	Components tested							Endpoint results		
Experiment	PGM (g/L)	Glucose (%)	Glutamine (mM)	$FeSO_4$ (μM)	Lincomycin (μg/mL)	EGTA (mM)	pH (units)	MHT* (log_2)	Bacterial density (OD/mL)	Final pH
1[a]	0.4	0.4	8.4	87.4	45.5	0.6	7.1	7.0	3.28	6.84
2[a]	1.1	0.6	10.4	162.7	45.5	0.6	7.1	6.75	4.73	6.78
3[b]	1.1	0.6	10.4	102.5	50.5	0.6	7.3	6.75	4.70	6.89
4[b]	1.1	0.6	10.4	102.5	50.5	0.6	7.3	7.25	4.66	6.90
5	1.1	0.6	20.0	78.9	42.7	1.0	7.3	6.25	5.23	7.18
6	1.1	0.1	13.7	126.9	58.6	0.7	8.5	7.5	1.32	8.32
7[b]	1.1	0.6	10.4	102.5	50.5	0.6	7.3	7.25	4.56	6.93
8[a]	1.1	0.6	16.5	87.4	45.5	0.6	7.1	7.5	4.96	6.94
9	1.1	0.6	0.5	126.9	58.6	0.7	6.0	3.75	3.51	4.04
10	1.1	1.0	7.2	78.9	81.8	0.4	7.3	7.0	4.20	5.46
11	0.1	0.3	7.2	78.9	81.8	0.4	7.3	6.75	3.63	4.85
12	1.1	0.6	10.4	102.5	50.5	0.1	6.0	5.0	3.67	4.45
13	2.0	0.3	7.2	78.9	42.7	1.0	7.3	6.25	4.75	4.86
14	0.1	0.3	16.8	55.4	34.8	0.4	7.3	5.0	3.78	4.89
15	1.1	0.6	10.4	5.0	58.6	0.7	8.5	6.75	4.43	7.12
16	0.1	0.8	13.7	126.9	58.6	0.7	8.5	6.75	3.72	5.93
17	1.1	0.6	10.4	102.5	50.5	0.1	8.5	6.0	3.32	6.97
18	1.1	0.6	20.0	173.2	34.8	0.4	7.3	5.25	6.62	7.25
19[a]	1.1	0.6	10.4	102.5	50.5	0.9	7.1	6.0	5.08	6.94
20	2.0	0.3	7.2	173.2	34.8	0.4	7.3	6.5	5.50	5.11
21	0.1	0.8	13.7	126.9	58.6	0.7	6.0	4.75	3.55	4.36
22[a]	1.7	0.4	8.4	87.4	45.5	0.6	7.1	7.75	3.80	7.08
23	0.1	0.3	7.2	173.2	34.8	0.4	7.3	6.25	3.70	4.97
24	2.0	0.8	13.7	126.9	58.6	0.7	6.0	6.5	4.31	4.56
25	1.1	0.6	20.0	78.9	81.8	0.4	7.3	8.5	4.98	7.16
26	1.1	1.0	16.8	55.4	34.8	0.4	7.3	6.75	5.06	5.33
27[b]	1.1	0.6	10.4	102.5	50.5	0.6	7.3	7.5	4.81	6.83
28[a]	1.1	0.8	8.4	87.4	45.5	0.6	7.1	6.75	5.71	6.55
29	1.1	1.0	7.2	173.2	34.8	0.4	7.3	6.75	4.85	5.28
30	2.0	0.3	7.2	78.9	81.8	0.4	7.3	8.0	4.87	5.15
31	2.0	0.8	13.7	126.9	58.6	0.7	8.5	7.75	5.30	6.82
32	1.1	0.6	10.4	200	81.8	0.4	7.3	9.0	4.11	6.69
33[a]	1.1	0.6	10.4	102.5	75.5	0.6	7.1	8.0	4.81	6.79
34	1.1	1.0	7.2	78.9	42.7	1.0	7.3	7.25	4.70	5.25
35	0.1	0.3	7.2	78.9	42.7	1.0	7.3	6.75	3.40	4.89
36	1.1	0.1	13.7	126.9	58.6	0.7	6.0	6.75	2.60	6.26
37	1.1	0.6	10.4	102.5	10.0	0.7	6.0	5.75	3.67	4.54
38	1.1	0.1	4.0	55.4	34.8	0.4	7.3	7.75	1.51	7.43
39	1.1	0.6	10.4	102.5	89.7	1.0	7.3	8.25	4.53	6.92
40	0.1	0.8	4.0	55.4	34.8	0.4	7.3	6.0	4.22	5.76
41	1.1	0.6	10.4	200	42.7	1.0	7.3	7.25	5.04	7.10
42	2.0	0.3	16.8	55.4	34.8	0.4	7.3	7.25	5.80	5.31
43	1.1	0.6	10.4	102.5	10.0	0.7	8.5	5.0	5.19	7.66
44	1.1	0.6	10.4	5.0	58.6	0.7	6.0	7.0	4.30	4.56
45	2.0	0.8	4.0	55.4	34.8	0.4	7.3	7.5	5.73	6.80
46[a]	1.1	0.6	10.4	102.5	50.5	0.6	8.3	7.5	5.21	7.43
47	1.1	0.6	0.5	126.9	58.6	0.7	8.5	7.5	4.16	7.12

[a]test points, [b]center points, *MHT indicates minimal hemagglutination titer detected by MRHA, associated with CFA/I surface expression
