# Supplementary material for: Identification of Novel Components Influencing Colonization Factor Antigen I Expression in Enterotoxigenic Escherichia coli
Source: PLoS One. 2015 Oct 30;10(10):e0141469. doi: 10.1371/journal.pone.0141469 (PMC4627747; doi:10.1371/journal.pone.0141469)
Supplement: S3 Table — (PDF) [file pone.0141469.s007.pdf]

Table S3. Significance and deviation of component effects on endpoints as determined by the Hadamard matrix

	CFA/I surface expression			Secreted LT			Bacterial density		
**Factor**	Coefficient[a]	St Dev [b]	p-val (%)[c]	Coefficient	St Dev	p-val (%)	Coefficient	St Dev	p-val (%)
Glucose	0.57	0.111	0.09	-0.116	0.116	34.2	0.238	0.016	< 0.01
Glycerol	0.13	0.113	29.2	-0.089	0.117	46.4	0.276	0.016	< 0.01
PGM	0.89	0.115	< 0.01	-0.391	0.117	0.85	0.287	0.016	< 0.01
Methionine	-0.06	0.111	62.9	-0.184	0.116	14.7	-0.007	0.016	65.5
Lysine	0.07	0.115	56.2	0.222	0.117	9.0	-0.132	0.016	0.02
Aspartic Acid	0.25	0.118	6.4	-0.075	0.117	53.4	0.077	0.016	0.27
Glutamine	0.47	0.115	0.34	0.071	0.117	55.8	0.066	0.016	0.55
Alanine	0.16	0.113	18.6	0.014	0.117	91.0	0.049	0.016	1.94
Leucine	-1.46	0.118	< 0.01	0.001	0.117	99.1	0.134	0.016	0.01
$MnCl_2$	-0.07	0.115	56.2	-0.228	0.117	8.3	0.085	0.016	0.16
$CaCl_2$	-0.03	0.115	81.5	-0.132	0.117	28.8	0.035	0.016	6.5
$ZnCl_2$	-0.55	0.111	0.11	-0.199	0.116	11.9	0.033	0.015	7.6
$FeSO_4$	0.54	0.118	0.17	0.038	0.117	75.3	0.140	0.016	0.01
Ammonia	-0.16	0.111	19.1	-0.040	0.116	73.7	-0.120	0.015	0.02
Bicarbonate	0.37	0.118	1.32	0.067	0.117	57.8	-0.144	0.016	< 0.01
Norepinephrine	-0.54	0.113	0.13	0.055	0.117	65.0	-0.069	0.016	0.45
Lincomycin	1.58	0.118	< 0.01	0.087	0.117	47.5	-0.127	0.016	0.02
cAMP	-0.40	0.115	0.80	0.047	0.117	69.5	-0.035	0.016	6.5
EGTA	0.40	0.115	0.80	-0.135	0.117	27.9	0.137	0.016	0.01
1,10 o-phen.	-0.81	0.120	0.02	0.224	0.116	8.5	-0.013	0.015	43.7

[a]Coefficients indicate the relative strength of the response, as shown in Fig 1.
[b]Standard deviation (St Dev)
[c]p-values <5% are considered significant and p-values from 5-10% at the limit of significance.
