# Supplementary material for: Identification of Novel Components Influencing Colonization Factor Antigen I Expression in Enterotoxigenic Escherichia coli
Source: PLoS One. 2015 Oct 30;10(10):e0141469. doi: 10.1371/journal.pone.0141469 (PMC4627747; doi:10.1371/journal.pone.0141469)
Supplement: S4 Table — (PDF) [file pone.0141469.s008.pdf]

**Table S4. Endpoint association with final pH using Spearman's correlates**

| Endpoint | Variable | Spearman ρho | p-val (%)* |
|---|---|---|---|
| Bacterial density (OD/mL) | Final pH | -0.3671 | 4.6 |
| Surface expressed CFA/I (MHT ($log_2$)) | Final pH | 0.1986 | 30.1 |
| Secreted LT (pg/OD) | Final pH | -0.0234 | 90.3 |

*p-values <5% are considered significant
