# Supplementary material for: Identification of Novel Components Influencing Colonization Factor Antigen I Expression in Enterotoxigenic Escherichia coli
Source: PLoS One. 2015 Oct 30;10(10):e0141469. doi: 10.1371/journal.pone.0141469 (PMC4627747; doi:10.1371/journal.pone.0141469)
Supplement: S5 Table — (PDF) [file pone.0141469.s009.pdf]

Table S5. Evaluation of component effects and interactions in SP1 media on CFA/I expression and bacterial density

A. CFA/I surface expression

	Component	Coefficient[a]	St. Dev.	p-val (%)*
Linear	**Lincomycin**	**1.382**	**0.212**	**< 0.01**
	**pH**	**0.924**	**0.210**	**0.104**
	**PGM**	**0.904**	**0.209**	**0.118**
	EGTA	0.352	0.212	12.6
	Glucose	-0.197	0.212	37.4
	Glutamine	0.118	0.213	59.1
	$FeSO_4$	-0.123	0.212	57.2
Squared	**pH**	**-2.374**	**0.462**	**0.032**
	**Glutamine**	**-1.081**	**0.460**	**3.84**
	PGM	-0.926	0.448	6.3
	EGTA	-0.864	0.443	7.7
	$FeSO_4$	0.754	0.441	11.5
	Glucose	0.639	0.463	19.5
	Lincomycin	0.172	0.447	70.8
	**Glutamine-Lincomycin**	**2.745**	**0.661**	**0.16**
	**Glutamine-pH**	**-2.020**	**0.641**	**0.92**
	**Lincomycin-pH**	**1.982**	**0.641**	**1.02**
	**$FeSO_4$-pH**	**1.888**	**0.641**	**1.33**
	**$FeSO_4$-Lincomycin**	**1.719**	**0.650**	**2.28**
	**PGM-EGTA**	**-1.420**	**0.643**	**4.93**
	**PGM-Glutamine**	**1.150**	**0.628**	**9.4**
	PGM-$FeSO_4$	-0.857	0.637	20.5
	PGM-Glucose	0.729	0.607	25.5
	Glucose-Lincomycin	-0.721	0.660	29.8
	Glucose-pH	0.690	0.641	30.5
	Glucose-EGTA	0.606	0.663	38.0
	PGM-pH	-0.512	0.641	44.1
	$FeSO_4$-EGTA	0.517	0.651	44.4
	Glucose-Glutamine	0.460	0.642	48.9
	Glutamine-EGTA	0.315	0.664	64.4
	PGM-Lincomycin	0.192	0.641	77.0
	EGTA-pH	0.112	0.641	86.5
	Glutamine-$FeSO_4$	-0.096	0.657	88.6
	Glucose-$FeSO_4$	0.095	0.654	88.7
	Lincomycin-EGTA	0.081	0.664	90.5

B. Bacterial density

	Component	Coefficient[a]	St. Dev.	p-val (%)*
Linear	**Glucose**	**0.387**	**0.055**	**< 0.01**
	**PGM**	**0.278**	**0.054**	**0.0340**
	**Glutamine**	**0.138**	**0.055**	**3.03**
	**Lincomycin**	**-0.104**	**0.055**	**8.6**
	EGTA	0.043	0.055	45.3
	pH	0.032	0.055	56.5
	$FeSO_4$	0.005	0.055	93.5
Squared	**Glucose**	**-0.747**	**0.121**	**< 0.01**
	**pH**	**-0.587**	**0.120**	**0.049**
	**PGM**	**0.239**	**0.117**	**6.6**
	$FeSO_4$	0.137	0.115	25.7
	Glutamine	0.110	0.120	37.8
	Lincomycin	0.090	0.117	45.7
	EGTA	-0.078	0.115	51.4
	**Glucose-pH**	**0.472**	**0.167**	**1.66**
	**$FeSO_4$-Lincomycin**	**-0.433**	**0.169**	**2.68**
	**Lincomycin-pH**	**-0.319**	**0.167**	**8.3**
	**Glutamine-Lincomycin**	**-0.310**	**0.172**	**9.9**
	Glutamine-EGTA	-0.210	0.173	25.0
	Glucose-Lincomycin	-0.201	0.172	26.8
	Glutamine-pH	-0.192	0.167	27.4
	Glucose-Glutamine	-0.185	0.168	29.3
	Glucose-$FeSO_4$	-0.149	0.171	40.1
	EGTA-pH	0.138	0.167	42.6
	$FeSO_4$-EGTA	-0.140	0.170	42.8
	PGM-pH	0.124	0.167	47.2
	PGM-Lincomycin	-0.110	0.167	52.4
	PGM-Glucose	-0.087	0.158	59.2
	PGM-EGTA	-0.062	0.168	71.8
	Glutamine-$FeSO_4$	-0.052	0.171	76.7
	PGM-Glutamine	0.044	0.164	79.5
	$FeSO_4$-pH	-0.033	0.167	84.8
	Lincomycin-EGTA	-0.007	0.173	96.9
	Glucose-EGTA	0.006	0.173	97.2
	PGM-$FeSO_4$	-0.003	0.166	98.5

[a]Coefficients indicate the relative strength of the response, which may be linear (top), squared (middle), or dependent on interactions (bottom).

*p-values <5% are considered significant while p-values from 5-10% are at the limit of significance (bold)
